# Lipidomics in Melanoma: Insights into Disease Progression and Therapeutical Targets

**DOI:** 10.3390/ijms27021040

**Published:** 2026-01-20

**Authors:** Vittoria Maresca, Emanuela Bastonini, Giorgia Cardinali, Enrica Flori, Daniela Kovacs, Monica Ottaviani, Stefania Briganti

**Affiliations:** Laboratory of Cutaneous Physiopathology and Integrated Center of Metabolomic Research, San Gallicano Dermatological Institute, IRCCS, 00144 Rome, Italy; vittoria.maresca@ifo.it (V.M.); emanuela.bastonini@ifo.it (E.B.); giorgia.cardinali@ifo.it (G.C.); enrica.flori@ifo.it (E.F.); daniela.kovacs@ifo.it (D.K.); stefania.briganti@ifo.it (S.B.)

**Keywords:** melanoma, lipidomics, metabolism, metastasis, biomarkers, therapy resistance, precision oncology, multi-omics, plasma lipid profiling, tumor microenvironment

## Abstract

Melanoma is the deadliest form of skin cancer, characterized by high metastatic potential and intrinsic heterogeneity. In addition to genetic mutations such as BRAF^V600E^ and NRAS, lipid metabolic reprogramming has emerged as a critical factor in tumor progression and therapy resistance. Lipid metabolism supports melanoma cell survival, phenotypic switching, immune evasion, and resistance to targeted therapies and immunotherapy, while also modulating susceptibility to ferroptosis. This review summarizes current knowledge on lipid dysregulation in melanoma, highlighting alterations in fatty acid synthesis, desaturation, uptake, storage, and oxidation, as well as changes in phospholipids, sphingolipids, cholesterol, and bioactive lipid mediators. These lipid pathways are tightly regulated by oncogenic signaling networks, including MAPK and PI3K–AKT–mTOR pathways, and are influenced by tumor microenvironmental stressors such as hypoxia and nutrient limitation. Advances in lipidomics technologies, particularly mass spectrometry-based approaches, have enabled comprehensive profiling of lipid alterations at bulk, spatial, and single-cell levels, offering new opportunities for biomarker discovery and therapeutic stratification. Targeting lipid metabolic vulnerabilities represents a promising strategy to improve melanoma diagnosis, prognosis, and treatment efficacy.

## 1. Introduction

Over the past few decades, the incidence of cutaneous melanoma (CM) has been steadily increasing worldwide, especially in Caucasian populations, transforming it into a malignancy of growing public-health concern. CM arises from the malignant transformation of neural crest-derived melanocytes in the epidermis and is the most lethal form of skin cancer, being responsible for around the 80–90% of all skin-cancer-related deaths globally [1]. According to GLOBOCAN 2022, melanoma constitutes ~1.7% of new cancer diagnoses but contributes disproportionately to mortality [2], with incidence peaking in Australia and New Zealand, ranging from 30 to 60 cases per 100,000 people annually [3,4]. Melanoma arises from a complex interplay of somatic and inherited genetic alterations that promote tumor initiation, progression, and heterogeneity. In most cases, CM is driven by acquired somatic mutations, most frequently activating changes in BRAF and NRAS genes, often associated with ultraviolet light (UV) exposure. Other recurrent somatic alterations involve genes such as PTEN, TP53, ARF, NF1 and KIT. In addition to these somatic events, hereditary factors contribute to melanoma susceptibility in approximately 10% of cases [5,6,7]. High-penetrance germline mutations in CDKN2A, CDK4 and BAP1 disrupt critical tumor-suppressor pathways, while variants in MC1R, encoding the α-melanocyte-stimulating hormone receptor, modulate pigmentation and are associated with UV-related mutagenesis; loss-of-function MC1R polymorphisms, commonly found in individuals with low phototype characterized by fair skin, red/blond hair, light-colored eyes, and freckling, further increase melanoma risk [8,9,10]. Epidemiological studies have identified a history of sunburns and intermittent solar exposure as key etiological factors for CM. Moreover, its incidence continues to rise particularly among populations with low phototype, and high UV exposure, either environmental or artificial [11,12]. Even childhood sunburn episodes are strong predictors of lifetime risk for CM [13]. Nonetheless, melanomas can also arise in non-sun-exposed areas (acral, mucosal), suggesting additional genetic and metabolic drivers [14,15]. Prevention strategies focus on UV protection, public-awareness campaigns, and dermatologic surveillance for early lesions. Despite advances in early detection and targeted therapies, melanoma remains a major public-health challenge due to its high metastatic potential, genetic heterogeneity, and ability to develop treatment resistance [16,17]. Early-stage CM confined to the primary site is curable by surgical resection, with 5-year survival exceeding 98%. However, this rate decreases sharply with dermal invasion and metastasis to distant sites such as regional lymph nodes, liver, brain, and lungs. At these stages, the disease becomes refractory to existing therapies [18]. Patients carrying MAPK/ERK mutations are treated with small-molecule BRAF/MEK inhibitors (e.g., vemurafenib, dabrafenib + trametinib), which target BRAF kinase with the V600E mutation and lead to rapid regression [19], although acquired resistance typically develops within six to ten months [20,21,22]. Many patients with metastatic melanoma are treated with immune checkpoint inhibitors (anti-PD-1, anti-CTLA-4) [23,24], which improve survival, though only about 45–50% respond [25]. Consequently, novel therapeutic interventions targeting alternative mechanisms are urgently needed to reduce drug-resistance. The multitude of molecular alterations observed in melanoma cells may be responsible for its aggressive nature, metastatic capacity, and substantial intra-tumoral heterogeneity. Melanoma exhibits the highest mutational burden among all cancers, primarily attributed to UV-induced photodamage [26]. However, this environmental insult triggers more than just genomic alterations; it fosters a chronic pro-inflammatory state and induces massive oxidative stress. Collectively, these factors force cells to undergo profound metabolic shifts, establishing the molecular framework for the substantial lipid profile changes characteristic of malignancy [27]. The synergy between high mutational load and persistent oxidative stress drives an intrinsic metabolic reprogramming that enables melanoma cells to adapt, survive, and develop resistance under fluctuating conditions [27,28,29]. Melanoma cells adapt dynamically between glycolysis and oxidative phosphorylation, shaping the availability of key intermediates for nucleotide, amino acid, and lipid synthesis. The Krebs cycle, largely fueled by glutamine, produces citrate, which is exported to the cytosol as the starting substrate for de novo lipid synthesis. Thus, changes in lipid composition reflect hallmark features of cancer-associated metabolic reprogramming [30,31] that occur faster than changes in proteins [32]. This highlights the need for a comprehensive understanding of melanoma metabolic reprogramming, particularly lipid metabolism, to improve diagnostic strategies, staging and therapeutic interventions. In this context, melanoma diagnosis remains complex and often subjective, relying on visual examination, dermoscopy, and histopathology. Partial biopsies can underestimate Breslow thickness, and melanomas may resemble benign nevi, complicating diagnosis, particularly in patients with numerous suspicious lesions [33]. There is increasing demand for more accurate, objective diagnostic tools to support pathologists, reduce errors, and lower healthcare costs [34,35,36]. Liquid biopsies represent a minimally invasive alternative, as blood carries molecules released by affected tissues. However, only a few serum-based biomarkers, such as lactate dehydrogenase or S-100 protein, are useful for monitoring disease course or recurrence [37,38]. Reliable biomarkers for melanoma diagnosis are still lacking.

Recent innovations in omics sciences significantly revolutionized the discovery of melanoma biomarkers. In particular, advances in analytical chemistry and computational biology have transformed lipidomics from a descriptive discipline into a powerful, quantitative, and spatially resolved approach for investigating the role of lipid profiles in cells, tissues, or biofluids, in skin cancer, including melanoma [39,40].

Since melanoma heterogeneity, progression, and therapeutic resistance are all connected and driven by lipid reprogramming, lipidomics can offer insights into melanoma initiation and progression, and improve the identification of biomarkers for diagnosis, prognosis, therapy monitoring, and resistance [41,42].

This review explores the key findings on lipid metabolism and how it interacts with genetic and metabolomic factors. It also looks at the important role of lipidomics in improving our understanding of melanoma and helping with diagnosis, staging and treatment.

## 2. Metabolic Dysregulation in Melanoma

Mutations in the BRAFV600E and NRAS oncogenes are among the most common genetic alterations in cutaneous melanoma and cause constitutive activation of the MAPK pathway, typically associated with growth factor-induced activation of tyrosine kinase receptors [43,44]. The MAPK pathway transfers signals from the membrane to the nucleus through a series of phosphorylations that culminate in the activation of ERK. Once in the nucleus, ERK initiates a vast program of metabolic reprogramming mainly orchestrated by c-MYC and assisted by HIF-1 in conditions of hypoxia. ERK also exerts a repressive action on MITF, which is a key regulator of melanocyte differentiation [30]. Metabolic reprogramming in melanoma supports rapid growth, survival, and metastatization of tumor cells and also confers high plasticity, allowing flexible switching between glycolysis and oxidative phosphorylation in response to genetic and environmental signals [45,46]. In this context, c-MYC extensively regulates glucose and glutamine metabolism. It acts synergistically with PI3K/AKT since AKT promotes GLUT1 translation and its transport to the plasma membrane [47,48]. c-MYC coordinates the expression of glycolytic enzymes and also increases the expression of glutamine transporters and glutaminase, allowing the production of glutamate and its subsequent entry into the tricarboxylic acid (TCA) cycle generating α-ketoglutarate [49]. In melanoma, glycolysis and the TCA cycle often function in a decoupled manner: glucose fuels glycolysis, while glutamine feeds the TCA cycle. Thus, the TCA cycle also has an anabolic value in transformed cells by providing precursors for the synthesis of nucleotides, amino acids and lipids [45,49]. The predominance of aerobic glycolysis leads to the production of large amounts of lactate, acidifying the tumor microenvironment and promoting metastasis and immunosuppression [30,50]. The BRAFV600E mutation amplifies the glycolytic phenotype by limiting mitochondrial biogenesis [30,51]. However, under metastatic or therapeutic stress conditions, cells can reactivate the axis between MITF and PGC1α, mainly involved in restoring a more efficient mitochondrial oxidative metabolism [52,53]. Glutamine is an essential resource for maintaining TCA intermediates and for supplying carbon and nitrogen, which are essential for the biosynthesis of macromolecules [54]. Variability in oxygenation within the melanoma mass induces distinct adaptations: in normoxia, nucleotide synthesis, lipogenesis, and high proliferation prevail; in hypoxia, nucleotide synthesis is blocked, cell division slows down, and lipids are oxidized to produce ATP [55,56]. The tumor microenvironment, typically hypoxic and acidic, is profoundly influenced by cancer metabolism. HIF-1α induces the expression of LDH and MCT1/4 transporters, facilitating the “lactate shuttle,” through which peripheral glycolytic cells produce lactate that deeper cells oxidize, establishing a true metabolic symbiosis [57,58]. The acidic, nutrient-impaired environment hinders the proliferation of T cells and the production of cytokines contributing to immune evasion and resistance to immunotherapy [30,52]. All these aspects confirm that metabolic reprogramming not only supports tumor growth, but is also a key element in metastasis, environmental adaptation, drug resistance, and evasion of immune control.

## 3. Lipid Dysregulation in Melanoma

Lipids are water-insoluble molecules broadly classified into fatty acids (FAs), triacylglycerides (TGs), phospholipids (PLs), and cholesterol (CH). Abundant in cellular organelles, they serve as key structural components of membranes. Beyond this, lipids play vital roles in energy storage, cell signaling, and as second messengers, while also contributing to cancer-related processes like immunoediting, angiogenesis, invasion, and migration [59,60,61]. The development of a lipogenic phenotype has been identified as an early biochemical hallmark of cancer cells [59,62,63,64,65].

Furthermore, FA synthesis is often upregulated in tumor cells, including melanoma cells, regardless of the availability of extracellular lipids [66]. Citrate, derived from the TCA cycle, is exported from mitochondria to the cytoplasm and converted into acetyl-CoA, initiating de novo lipogenesis (DNL), a recognized hallmark of cancer metabolism [62,67]. This process remains constitutively active in many tumors, supplying most of the intracellular lipids required for rapid cell growth and survival [68]. At the level of acetyl-CoA, lipid synthesis diverges thanks to the action of 3-hydroxy-3-methyl-glutaryl-coenzyme A reductase (HMGCR), the acetyl-CoA is converted into mevalonate and the synthesis of cholesterol takes place. Alternatively, acetyl-CoA can be converted into malonyl-CoA by acetyl-CoA carboxylase 1 (ACC1), representing the first committed step in FA synthesis. FA synthase (FASN) then catalyzes the conversion of malonyl-CoA into palmitic acid. This primary FA can subsequently be elongated and desaturated to generate a variety of saturated and unsaturated fatty acids, which are crucial for supporting cancer cell growth, membrane biosynthesis, and signaling functions. In addition to DNL, the FA content in cancer cells is influenced by uptake and fatty acid oxidation processes [67]. Besides FAs, all complex lipid species, such as PLs, their derivatives, like bioactive lipids, and products of partial oxidation participate in maintaining a dynamic equilibrium that supports the metabolism and the signaling of the transformed cell. Therefore, alterations in lipid metabolism may be considered a crucial element of melanoma biology, complementing the roles of glucose and glutamine. In this regard, studies assessing the metabolic behavior of melanoma have demonstrated that such phenotypic plasticity confers adaptive advantages that favor proliferation and survival [63,64,65].

### 3.1. Fatty Acid: Role in Melanoma and Therapeutic Targets

#### 3.1.1. De Novo Lipogenesis

DNL enables cells to create new, strategically structured biomass that can promote melanoma growth by supplying FAs for membrane synthesis, signaling molecules, and energy storage. These reactions are catalyzed by ATP citrate lyase (ACLY), ACC, and FASN, which are upregulated downstream of the PI3K–AKT–mTOR and BRAF–MEK–ERK pathways [62]. In human melanoma, the expression of ACC1 and FASN is higher compared to that observed in common nevi [69,70]. Interestingly, an increase in FASN expression occurs regardless of BRAF and NRAS mutation status, but it is associated with greater Breslow thickness and a poorer prognosis [71]. Palmitic acid can be elongated by elongases (ELOVLs) and further modified by stearoyl-CoA desaturase (SCD). This enzyme catalyzes the conversion of saturated fatty acids to D9-monounsaturated fatty acids (MUFAs), a process that is crucial for maintaining membrane fluidity and protecting cancer cells from lipid oxidative stress and peroxidation, including that caused by chemotherapy [72,73]. The primary substrates of SCD are palmitoyl-CoA and stearoyl-CoA, which produce palmitoleoyl-CoA and oleoyl-CoA, respectively [74,75]. In several types of cancer cells, SCD1 plays a crucial role in regulating resistance to ferroptosis, which is a type of iron-dependent cell death caused by the accumulation of oxidized polyunsaturated fatty acids (PUFAs) in cell membranes [76,77,78,79]. Key enzymes in this process include glutathione peroxidase 4 (GPX4), which neutralizes lipid peroxides, and acyl-CoA synthetase long-chain family member 4 (ACSL4), which incorporates PUFAs into membranes. Disruption of this balance can trigger ferroptosis [80]. The ratio of saturated/monounsaturated fatty acids (SFAs/MUFAs) also influences lipid peroxidation and ferroptosis. MUFA production, driven by SCD1 and regulated by sterol regulatory element-binding protein-1 (SREBP1), protects cancer cells from ferroptosis since MUFAs are resistant to lipid peroxidation [81]. Furthermore, increased amount of oleic acid and its activation by ACSL protects melanoma cells from ferroptosis and boosted their capacity to form metastasis [82]. Growth and lipid metabolism are closely interconnected through transcriptional regulation governed by SREBP-1 [83,84]. It is now well established that common genetic mutations, such as those in p53 or PTEN [85,86] and aberrant growth factor signaling pathways, including ERK1/ERK2 MAPKs triggered by EGF, HER2, KGF [87,88,89,90] can enhance SREBP1 activity. This leads to the upregulation of lipogenic genes, contributing to metabolic reprogramming in cancer cells. FA synthesis is stimulated by oncogenic pathways such as the PI3K–AKT–mTORC1 axis and MYC, primarily through the activation of SREBP1 [91,92]. This leads to increased production of MUFAs, which helps protect cells from lipotoxicity and endoplasmic reticulum (ER) stress. A study on melanoma cells confirmed that DNL depends on the proteolytic activation of SREBP-1 and demonstrated that this process is a key mediator of the oncogenic effects of the BRAF gene, contributing to therapy resistance. Specifically, it was found that while BRAFv600E-targeted therapy inhibits lipogenesis in therapy-sensitive cells, resistant cells consistently restore SREBP-1 activation and lipogenesis [93]. FAs support not only lipid metabolism, but also the regulation of protein function through acylation [94]. For instance, the S-palmitoylation of MC1R, which involves the covalent attachment of palmitic acid to cysteine residues on the protein, enhances its activity and has been linked to reduced melanoma development in mice [95]. Elevated SCD1 expression has been detected in various types of cancer and is associated with greater tumor aggressiveness and a poorer prognosis for patients. Studies indicate that SCD1 promotes cancer progression by encouraging cell proliferation, migration, metastasis, and tumor growth [96].

Transformed and cancerous tissues often exhibit elevated levels of MUFAs [97,98]. Cancer cells that are growing quickly need more MUFAs, particularly for synthesizing components of cell membranes like PLs, TGs, and cholesterol esters (CEs) [87]. Tumors from different tissues show increased MUFA-rich lipids (especially phosphatidylcholine) alongside reduced SFAs and PUFAs. This buildup is closely related to higher levels of SCD1 expression in cancer cells [98,99]. In addition, the process of epithelia-mesenchymal transition (EMT), which has been demonstrated to be implicated in the acquisition of invasive and metastatic potential of cancer cells and the subsequent decrease in patient survival, is also accompanied by significant changes in lipid metabolism [100,101]. Melanoma cells show high plasticity, allowing them to shift states during tumor progression. Under stress (e.g., hypoxia, low glucose, inflammation), MITF expression decreases, promoting a more invasive, stem-like, EMT-associated phenotype [102]. These MITF-low expressing cells spread and later revert to high MITF expression at metastatic sites to support growth [103]. Fatty acid metabolism regulates melanoma progression by influencing MITF expression. Low activity of enzymes like ACLY and SCD reduces MITF levels [104], promoting a de-differentiated, invasive state [105]. In addition, low SCD activity leads to ER stress and inflammatory signaling (Via ATF4 and NF-κB), further suppressing MITF and enhancing melanoma cell plasticity and metastasis.

EMT has been also associated with the presence of cancer stem cells (CSCs) [106]. CSCs are an uncommon subpopulation of malignant cells that are intrinsically resistant to cytotoxic therapies [107]. This leads to the expansion of CSCs as they attempt to replace dying tumor cells. Maintenance of CSCs has been reported to result from an altered metabolic status characterized by a larger pool of MUFAs generated by SCD1 activity [108,109]. SCD1 has been shown to regulate stemness in 3D spheroid cultures of melanoma cell lines by increasing MUFA levels and upregulating stem cell markers such as OCT4, NANOG, CD133, and SOX2 [110]. Furthermore, this experimental model has revealed that higher levels of SCD1 expression are associated with melanoma growth and spread. It has also been linked to the ability of CSCs to resist BRAF/MEK inhibition [110]. In addition, upregulation of SCD1 suppresses ferroptosis [111].

Alongside changes in fatty acid desaturation, increased expression of ELOVL2 is crucial for melanoma cells to establish brain metastases [112]. Mechanistically, ELOVL2 supports endoplasmic-reticulum homeostasis and inhibits apoptosis by elongating essential fatty acids to produce PUFAs.

#### 3.1.2. Targeting Fatty Acid DNL and Inducing Ferroptosis

Pharmacologic inhibition of lipogenic enzymes has demonstrated antitumor activity in preclinical models. FASN inhibitors (e.g., TVB-2640, IPI-9119) suppress melanoma cell proliferation, induce endoplasmic-reticulum stress, and promote apoptosis [113]. In combination with BRAF inhibitors (vemurafenib or dabrafenib), FASN blockade delays the onset of resistance by restricting lipid supply needed for adaptive growth [114,115]. Similarly, ACC inhibitors such as ND-646 impair the conversion of acetyl-CoA to malonyl-CoA, reducing fatty acid synthesis and enhancing oxidative stress [116]. The treatment with MF-438, an SCD1 inhibitor, was found to selectively target cancer cells while sparing non-transformed cells. The aforementioned inhibition selectively killed CSCs and partially restored sensitivity to the combination of BRAF and MEK inhibitors, such as vemurafenib and trametinib [110]. Inhibiting FASN or SCD1 induces ER stress, ferroptosis, and apoptosis [117]. Although ferroptosis is crucial in cancer progression and impacts the success of immunotherapy, the precise molecular mechanisms governing the process remain largely unclear in melanoma cells. Wnt/β-catenin signaling pathway exerts a regulatory influence on both ferroptosis and the efficacy of immunotherapy in melanoma, by modulating MITF and its downstream targets, PGC1α and SCD1. Inhibiting this pathway has been shown to increase lipid peroxidation, enhance ferroptosis, and improve the response to anti-PD-1 immunotherapy in melanoma models [118]. Pharmacologic inhibition of SCD1 (e.g., CAY10566, A939572) re-sensitizes melanoma cells to targeted therapies and induces ferroptotic cell death [96]. Combining SCD1 inhibitors with ferroptosis inducers, such as erastin or RSL3, synergistically enhances tumor killing in vitro and in vivo [119,120]. These results emphasize the importance of lipid desaturation in maintaining redox homeostasis and making it a promising metabolic target.

Melanoma cell lines derived from patients and induced into a mesenchymal state Via TGF-β treatment show resistance to BRAF inhibitors like vemurafenib but are sensitive to the ferroptosis inducer RSL3 [79]. Interestingly, while metastasis-derived cell lines are resistant to GPX4 inhibitors, those in a mesenchymal state remain vulnerable [121]. This underscores the role of lipid metabolism reprogramming and GPX4-mediated ferroptosis suppression in metastatic cells. Given GPX4’s ability to block lipid peroxidation and ferroptosis, it emerges as a promising therapeutic target [122]. Consequently, GPX4 inhibitors are being actively investigated for their potential in treating various cancers [123,124,125].

While in vitro studies suggest that these compounds could represent a promising strategy, especially in cases of melanoma resistance that do not respond to standard immunotherapy protocols, the use of compounds capable of modulating lipid pathways at various levels has not yet entered the therapeutical practice for melanoma. At the present, phase 1 and 2 studies on advanced solid tumors have shown that the clinical application of FASN inhibitors is limited by significant challenges, primarily related to ‘on-target’ side effects and metabolic compensatory mechanisms [126,127]. In parallel, however, an approach based on omics technologies and associated bioinformatics, conceived as a tool that is already concretely available, is becoming increasingly important. Rather than focusing exclusively on direct pharmacological modulation, high-resolution omic profiling allows for the analysis of key genes, such as FASN, to predict responsiveness to treatments. The identification of spontaneous mutations or specific molecular signatures enables the a priori stratification of patients, serving as a reliable biomarker not only for immunotherapy but potentially also for anticipating the efficacy of chemotherapeutic treatments. Consequently, this leads to higher responsiveness to immune checkpoint inhibitors [128,129]. Ultimately, while direct metabolic modulation remains an experimental and complex field, the integration of metabolic and genetic data through bioinformatics provides a solid framework for optimizing patient selection and guiding clinical decisions within the scope of precision oncology.

#### 3.1.3. Fatty Acid Oxidation

Fatty acid oxidation (FAO) plays a vital role in maintaining ATP production and nicotinamide adenine dinucleotide phosphate (NADPH) balance, both essential for cancer cell growth and survival. AMP-activated kinase (AMPK) plays a key role in helping cancer cells adapt to stress by triggering a catabolic shift that boosts the levels of ATP and NADPH [130]. Melanoma cells exhibit a high degree of energetic demand, and mounting evidence indicates that they consume FAs from the tumoral microenvironment by activating FAO [131]. The FAO process is known to be triggered under conditions of oxidative stress. The process is initiated by the activation of FFAs, which results in the generation of fatty acyl-CoA by ACSLs [132]. Next, acyl-CoA is converted to acylcarnitine by the enzyme carnitine palmitoyltransferase 1 (CPT1). The subsequent steps involve the transport of acylcarnitine into the mitochondria Via carnitine acylcarnitine translocase (CACT) and the reconversion of acylcarnitine to acyl-CoA by CPT2. This process enables acyl-CoA to enter the FAO cycle and subsequently the Krebs cycle, facilitating energy production. Such activation is a physiological response that is intended to maintain ATP production and redox homeostasis [132]. An increased expression of FAO enzymes was found to confer metastatic and drug-resistant phenotypes and correlate with a poor overall survival in melanoma patients [130,133]. The expression of the genes encoding the key enzymes CPT1A and CPT2 is increased in melanoma [134]. This enhances FA transport and supports the production of NADPH, which reduces oxidative stress. FAO is further boosted by MAPK inhibitor treatment, which upregulates FA transporters, peroxisome proliferator-activated receptor alpha (PPAR-α), and CPT1A [135]. This highlights a metabolic adaptation linked to melanoma progression and therapy resistance.

How FAO supports melanoma progression remains not fully understood. It is hypothesized that FAs provide a key source of acetyl-CoA, fueling citrate production Via the TCA cycle and supplying ATP, especially under nutrient-deprived conditions [130]. Notably, studies involving co-culture of melanoma cells with adipocytes revealed that tumor cells use adipocyte-derived lipids for FAO, reducing reliance on de novo lipogenesis [136,137]. Interestingly, glucose metabolism remained unchanged, suggesting that glycolysis continues independently of FAO activity [138].

#### 3.1.4. Lipid Uptake and Storage

Lipolysis, the process of breaking down complex lipids into free fatty acids (FFAs), and their subsequent uptake, plays a substantial role in increasing the cellular FFA pool [66,139]. Cells can synthesize FAs de novo. However, they can also acquire them from extracellular sources. Recent studies have demonstrated a correlation between alterations in lipid uptake proteins and the tumor cells adaptability, as well as drug resistance promotion [140]. Increased expression of lipid transporters facilitates fatty acid uptake, contributing to cancer metastasis by enhancing tumor cell plasticity [141,142]. This phenomenon underscores the capacity of cancer cells to modulate their behavior in accordance with energy demands and microenvironmental signals. Melanoma cells also acquire lipids from the microenvironment, particularly from adipocytes, Via transporters such as FA translocase (FAT/CD36), plasma membrane-associated FA binding proteins (FABP) or FA transport proteins (FATP CD36). These lipids, which fuels β-oxidation promote epithelial–mesenchymal transition, enhancing metastatic potential. Recent findings show that melanoma cells exposed to lipids secreted by aged fibroblasts upregulate FATP2, increasing FA uptake [143]. Additionally, FABP7 has been linked to heightened proliferation and invasiveness in melanoma cells [144,145]. CD36, another FA transporter, is highly expressed in metastasis-initiating melanoma cells and correlates with poor prognosis, emphasizing the key role of FA uptake in melanoma progression [146]. Notwithstanding the advantages associated with lipid uptake, an accumulation of free FAs can result in lipotoxicity [147]. The presence of extracellular oleic acid in the lymph promotes melanoma spread through the lymphatic system and supports metastasis by preventing ferroptotic cell death in melanoma cells that are experiencing high levels of oxidative stress [82]. In order to counteract this cytotoxic mechanism, cells regulate lipid availability by storing fatty acids as neutral lipids, such as CE and triacylglycerols (TAGs), inside lipid droplets (LDs) [148,149]. LDs are dynamic organelles that function as a storage for FA, based on cellular needs. They consist of a core of neutral lipids encased in a phospholipid monolayer. In the context of tumor cells, these elements fulfill a protective function by impeding lipid accumulation and attenuating oxidative stress [149]. If needed, TAGs are subject to sequential hydrolysis by three distinct lipases, resulting in the release of FFAs. These FFAs can then be mobilized for various purposes, including energy production, membrane synthesis, and the generation of essential lipid-derived molecules. Furthermore, LDs have been identified as sites for the synthesis of lipid mediators, including oxylipins and lysophospholipids, which have been associated with the promotion of tumor progression [150]. LDs interact with mitochondria and peroxisomes to coordinate lipid utilization, in particular acting as a shuttle of FAs to the mitochondria for β-oxidation [151]. Their accumulation correlates with dedifferentiation, metastasis, and therapy resistance [152]. The process of LDs accumulation has been demonstrated to be induced by hypoxia through a HIF-1α-dependent mechanism and is linked to enhanced FA uptake, which is mediated by FABPs [153]. A lipid-storing phenotype, characterized by LDs accumulation, has been demonstrated in a melanoma stem cell model in comparison to more differentiated melanoma cells [154]. A link was found between the melanocytic state of tumor cells and their dependence on LDs in a zebrafish melanoma model [155]. This state is also conserved in human cells and tumors and is characterized by increased FA uptake, a greater number of LDs, and reliance on FA oxidative metabolism. Disrupting LDs formation inhibits cell cycle progression and slows melanoma growth, revealing a metabolic vulnerability that could be targeted for therapeutic purposes.

#### 3.1.5. Therapeutic Approaches Inhibiting Fatty Acid Uptake and Oxidation

Melanoma cells can scavenge exogenous fatty acids from the surrounding microenvironment, particularly under metabolic stress or therapy exposure. The lipid transporter CD36 mediates uptake of long-chain fatty acids from neighboring adipocytes and promotes metastatic dissemination [156]. CD36 blockade or dietary lipid restriction limit metastatic colonization and enhance the response to immunotherapy in preclinical models [157]. Similarly, inhibition of FAO through CPT1A antagonists (e.g., etomoxir) impairs ATP generation and redox buffering in drug-resistant melanoma cells. FAO suppression also restores sensitivity to BRAF/MEK inhibitors and induces metabolic crisis under nutrient limitation [158]. Nevertheless, systemic FAO inhibition must be approached cautiously due to cardiac toxicity, underscoring the need for melanoma-selective intervention strategies [159]. A recent study hypothesized that targeting key convergent subnetworks within the MAPK and PI3K pathways could eliminate cancer cells while reducing toxicity [160]. A detailed analysis of signaling in MAPKi-resistant NRAS-mutant melanoma revealed a critical subnetwork involving S6K1/2 and PPARα, essential for lipid metabolism in cancer. In MAPKi-sensitive cells, S6K1/2 is controlled by both MAPK and PI3K pathways, whereas in resistant cells, regulation occurs primarily through PI3K. Additionally, the study found that selectively depleting S6K2, thereby uncoupling S6K1 and S6K2, induced a lipid metabolic imbalance characterized by ER stress and lipid peroxidation. This disruption led to cell death, particularly in MAPKi-resistant NRAS-mutant melanomas. A comparative analysis of LDs composition showed that the dedifferentiated melanoma cell line M381 had higher levels of unsaturated CEs and TAGs compared to more differentiated melanoma lines [161]. Notably, prolonged treatment with the SCD1 inhibitor CAY10566 reduced unsaturated lipid levels in LDs and caused an accumulation of saturated fatty acids, which can alter membrane fluidity and trigger apoptosis [161].

### 3.2. Cholesterol Role in Melanoma and Therapeutic Targets

#### 3.2.1. Cholesterol Synthesis and Metabolism

Sterols are a subgroup of isoprenoids, with CH being the predominant sterol found in mammalian tissues. CH is essential for maintaining membrane integrity and fluidity. As a key component of lipid rafts, it also influences endocytosis, membrane trafficking, cell signaling, and motility. CH and FA synthesis both originate from acetyl-CoA but are regulated by different SREBPs: FA synthesis (DNL) depends on SREBP1a and SREBP1c, while CH biosynthesis is mainly controlled by SREBP2 [162]. CH is synthesized through the mevalonate pathway [163], whose enzyme expression is regulated by the transcription factor SREBP2. The process starts with the condensation of three acetyl-CoA molecules to form HMG-CoA, which is then reduced by HMGCR, the rate-limiting enzyme, to generate mevalonate. Mevalonate is further processed into farnesyl pyrophosphate, a crucial intermediate used for protein prenylation through geranylgeranyl pyrophosphate and for cholesterol synthesis Via squalene formation.

Melanocytes regulate CH levels through de novo synthesis Via HMGCR, but they can also incorporate cholesterol from outside the cell through the low-density lipoprotein receptor (LDLR)/Apo-B100 pathway [164]. High intracellular cholesterol suppresses SREBP2 activation and switches on liver X receptors (LXRs), reducing CH production and uptake while promoting CH removal [165]. Excess CH is converted by acyl-CoA-cholesterol acyltransferases (ACATs) into less toxic cholesteryl esters for storage in lipid droplets or excretion. In cancer cells, including melanoma, CH homeostasis is disrupted, contributing to tumor progression by enhancing proliferation, migration, and invasion [166]. This dysregulation involves increased cholesterol biosynthesis, elevated LDLR-mediated uptake, enhanced esterification Via ACAT1, and higher oxysterol production [167]. In melanoma, activation of the SREBP pathway and its positive feedback with PI3K-AKT-mTORC1 signaling supports tumor growth both in vitro and in vivo [168].

Oxysterols, oxidized derivatives of cholesterol, are abundantly present in the tumor microenvironment (TME) and play significant roles in modulating cancer progression, immune response, and cell signaling. Oxysterol 27-hydroxycholesterol has the capacity to stimulate the growth of melanoma cells by sustaining the activity of the AKT/MAPK signaling pathway [169]. In contrast, pharmacological activation of LXRβ, the predominant LXR isoform in melanoma cells, significantly suppresses tumor invasion and metastasis [170]. Collectively, these findings underscore a robust correlation between dysregulated cholesterol metabolism and the progression of melanoma.

CH and SLs are key components of lipid rafts, membrane domains that host critical signaling pathways for cancer survival and metastasis [171]. CH depletion using agents like methyl-cyclodextrin (MCD) inhibits melanoma progression by disrupting AKT signaling Via Src kinase inactivation and PP2A phosphatase reactivation, inducing apoptosis, altering cell morphology, and impairing migration [172,173,174]. MCD also blocks integrin internalization and focal adhesion turnover. Additionally, CH depletion reduces V-ATPase activity, lowering the acidity of the TME and impairing protease function, ultimately limiting melanoma cell invasion and metastasis [175,176].

#### 3.2.2. Targeting Cholesterol Pathway

Targeting cholesterol metabolism has emerged as a promising anticancer strategy. Statins, which inhibit HMGCR, have demonstrated the capacity to enhance the efficacy of immunotherapy and reduce cancer-related mortality across a range of tumor types, including melanoma [129]. Their potential as anti-tumor agents has also been explored in clinical trials. Despite their potential, the use of statins in cancer therapy remains controversial. Meta-analyses of clinical trials have shown that adding statins to systemic anticancer treatments did not significantly improve overall or progression-free survival in patients with solid tumors [177,178]. However, a number of findings suggest potential benefits in melanoma, considering that statin use has been linked to reduced Breslow thickness [179] and recent studies indicate a possible reduction in recurrence rates among patients with high-risk, ulcerated primary melanoma [180].

Inhibiting SOAT (acyl-CoA:cholesterol acyltransferase) has been demonstrated to reduce tumor growth and enhance immune checkpoint blockade efficacy [181,182]. SOAT is the enzyme that converts free CH into CH esters, which accumulate in lipid droplets and protect against ferroptosis.

**Figure 1:**
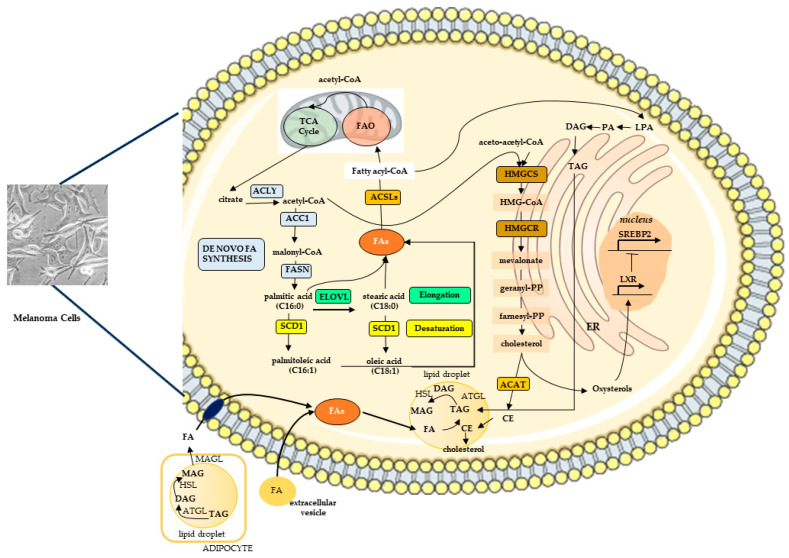
Schematic overview of the principal pathways related to the synthesis and me tabolism of fatty acid and cholesterol involved in melanoma. (Adapted from [41]) Enzymes: ACAT (acyl-CoA-cholesterol acyltransferase); ACC (acetyl-CoA carboxylase); ACLY (ATP citrate lyase); ACSL (acyl-CoA synthetase long-chain family member); ELOVL (fatty acid elongase); FASN (FA synthase); HMGCR (3-hydroxy-3-methyl-glutaryl-coenzyme A reductase); HMGCS (3-hydroxy-3-methyl-glutaryl-coenzyme A synthase); SCD1 (stearoyl-CoA desaturase1); Receptors and Transcription Factors: LXRs (liver X receptors); SREBP1 (sterol regulatory element-binding protein-1); Metabolic Pathways: TCA (tricarboxylic acid); FAO (Fatty acid oxidation); Lipid Species: MAG (monoacylglycerol); DAG (diacylglycerol); TAG (triacylglycerol); FA (fatty acid); CE (cholesterol ester); LPA (lysophosphatidic acid); PA (phosphatidic acid).

The principal pathway involved to the synthesis and metabolism of FAs and CH are summarized in Figure 1

### 3.3. Phospholipids: Role in Melanoma and Therapeutic Targets

Glycerophospholipids (GPLs) and certain sphingolipids (SLs) are the main classes of phospholipids in cells. GPLs are built on a glycerol backbone, while SLs contain a sphingoidbase derived from serine and a long-chain fatty acyl-CoA. Both are key structural components of cell membranes and precursors to signaling molecules [183].

#### 3.3.1. Glycerophospholipids Synthesis and Metabolism

GPLs specifically consist of 1,2-diacylglycerol linked to various polar headgroups Via a phosphodiester bond, forming distinct types like phosphatidylcholine (PC), phosphatidylserine (PS), phosphatidylethanolamine (PE), phosphatidylinositol (PI), and phosphatidylglycerol (PG). The process of forming new GPLs starts with the addition of FAs to PA, a key building block. The main pathway for making PC, the most abundant GPL, is the Kennedy pathway. This pathway has three enzymatic steps: choline is phosphorylated by choline kinase, CDP-choline is formed Via CTP:phosphocholine cytidylyltransferase, and choline is transferred to diacylglycerol (DAG) through CDP-choline:1,2-diacylglycerol cholinephosphotransferase to form PC [184]. The second most abundant phospholipid is PE, which can be synthesized de novo or formed through a head group exchange reaction from PS. It has been demonstrated that cancer cells undergoing to EMT exhibit increased PC content. Furthermore, PA has been shown to support cancer stemness by suppressing apoptosis [185]. Notably, melanoma-derived microvesicles have the capacity to promote metastasis in a PS-dependent manner, likely by suppressing the host’s inflammatory immune responses [186]. Lipidomic analyses revealed altered GPL metabolism in zebrafish V12RAS-driven melanoma, with elevated levels of PE and PC species [187]. Primary melanoma samples have higher total PC and PG levels than nevus melanocytes [188]. Mass spectrometry showed increased levels of PC, PG, PA, and PI in both primary and metastatic melanoma cells [189]. Phosphatidylinositol-3′-kinases (PI3Ks) are a class of lipid kinases that phosphorylate the 3′ hydroxyl group on the inositol ring of PIs. This enzymatic activity generates key signaling molecules, such as PI(3,4,5)P3 and PI(3,4)P2, which activate downstream pathways involved in vital cellular functions including cell cycle, programmed cell death, cell proliferation, angiogenesis, membrane dynamics, autophagy, and metabolic processes in both healthy and tumor cells. Activation of PI3K-lipid signaling is at least partly responsible for the transformation of benign nevi into malignant melanoma, highlighting its crucial role in early melanomagenesis [190]. In melanoma, the oncogenic effects of the PI3K pathway appear to be mediated primarily through RAC signaling rather than the canonical AKT pathway [191]. This is supported by evidence showing that inhibiting AKT or mTOR, either pharmacologically or genetically, fails to suppress cell growth or induce cell cycle arrest, even in the presence of elevated pAKT levels across melanoma cell lines. The conversion of normal melanocytes into metastatic melanoma cells involves multiple cooperating events, with the constitutive activation of PI3K-lipid signaling playing a pivotal role in melanoma progression. In fact, up to 70% of melanomas harbor oncogenic alterations in PI3K-lipid signaling that result in increased levels of PI(3,4,5)P3 [192,193]. In preclinical models, the activation of the PI3K-AKT signaling pathway has been observed to synergize with alterations, such as the expression of the BRAFV600E oncoprotein kinase, to promote melanoma progression and metastasis [194]. GPL composition is regulated not only by synthesis and head group exchange but also by the Lands’ cycle, a remodeling process involving deacylation and reacylation [195]. Lysophosphatidylcholine acyltransferase (LPCAT) enzymes are central to this process, helping to maintain lipid homeostasis by modulating PC species in various tissues. Melanoma cells show a high capacity to hydrolyze lysophosphatidylcholine (LPC), produced from PC by phospholipase A2 (PLA2) [196]. While LPC has anti-metastatic effects, disrupting focal adhesions, impairing integrin function, and limiting spread autotaxin (ATX) can convert LPC into lysophosphatidic acid (LPA) [197]. Elevated ATX levels in melanoma promote cell motility and invasiveness, increasing metastatic potential in vivo [198]. Moreover, LPA, generated through the PLA2-catalyzed deacylation of PA, has been shown to favor melanoma invasion in both 2D and 3D models [199]. Additionally, LPA was reported to induce degradation of MITF [200]. Sterols are a subgroup of isoprenoids, with CH being the predominant sterol found in mammalian tissues. CH is essential for maintaining membrane integrity and fluidity.

#### 3.3.2. Sphingolipids Synthesis and Metabolism

SLs consist of an 18-carbon amino alcohol backbone, usually sphingosine, linked to a fatty acid by an amide bond and a headgroup at the primary hydroxyl. These molecules range from simple forms like ceramide (CER), sphingosine (SPH), and sphingosine-1-phosphate (S1P) to more complex types such as sphingomyelin (SM), glycosphingolipids, and gangliosides. Numerous studies have shown that SL metabolism is altered in melanoma cells, leading to reduced intracellular levels of ceramide, a molecule known to trigger apoptosis [41]. CERs and its derivatives are involved in key biological processes such as cancer initiation, progression, and the response of tumor cells to chemotherapy and radiation. These observations highlight the pivotal role of ceramide metabolism in melanoma pathogenesis. One of the mechanisms through which CERs are synthesized involves the hydrolysis of membrane SMs. The expression of acid sphingomyelinase, the enzyme responsible for hydrolyzing SM into CER, decreases progressively from benign nevi to primary melanomas and further in metastases [201]. Its activity is lower in hyperpigmented melanomas compared to hypopigmented ones, suggesting an inverse relationship with melanin content. In the context of melanoma, there is a documented tendency of enzymes responsible for ceramide production to exhibit low activity. This phenomenon results in the preferential consumption of CERs, leading to the production of S1P and gangliosides [202,203,204]. The delicate balance between CER and S1P levels, referred to as the CER-S1P rheostat, plays a crucial role in determining cancer cell fate, promoting either proliferation or cell death. Therapeutic strategies aiming to shift this balance toward CER accumulation or S1P inhibition may effectively induce cancer cell death. CERs include over 200 distinct molecules generated Via de novo synthesis or cleavage of membrane precursors [205].The presence of low ceramide synthase 6 (CerS6) expression in melanoma cells has been correlated with malignant behavior, as evidenced in human melanoma cell lines WM35, WM451, and SKMEL28 [206]. Silencing CerS6 in melanoma cell lines led to increased proliferation and invasion. This downregulation also suppressed WNT5A, a growth factor that typically promotes melanoma cell proliferation and invasion. These findings suggest that CerS6 functions as a tumor suppressor in melanoma, and its overexpression may offer a potential therapeutic strategy.

CERs are broken down by five types of ceramidases, each with unique features and preferred substrates [207]. Among these ceramidases, acid ceramidase (AC), encoded by the gene ASAH1, is particularly significant due to its potential role in cancer progression and resistance to therapy [208]. AC is highly expressed in melanocytes and in proliferative melanoma cells, as well as in stage II melanoma biopsies from human subjects. However, despite high levels of AC, melanoma cells have reduced CER production, which is likely due to suppressed expression of key enzymes of de novo CER biosynthesis [204].

Downregulation of AC is associated with the transcriptional reprogramming of proliferative melanoma cells into an invasive cell subpopulation. ASAH1 inhibition initially causes an increase in CERs and a reduction in S1P, then S1P production increases significantly, through the action of sphingosine kinase 1 (SphK1), triggering the loss of E-cadherin and raising ganglioside levels [209]. This promotes EMT and enhances adhesion to the extracellular matrix, contributing to the aggressive behavior of melanoma.

SphK1 converts sphingosine into S1P, a bioactive sphingolipid that promotes tumor growth. SphK1 shows increased expression and/or activity in melanoma cells compared to normal melanocytes, in both cell lines and human tissue samples [210,211,212]. This suggests a shift in the S1P–CER balance toward S1P in melanoma. This is supported by the fact that the SGPL1 gene, encoding S1P lyase (SPL), which degrades S1P, is downregulated in melanoma cell lines relative to melanocytes, indicating its possible suppression during melanoma development [213]. S1P inhibits drug-induced apoptosis in melanoma cells, an effect reversible by C6-ceramide pre-treatment [214]. Silencing SK1 Via siRNA reduces melanoma cell proliferation and promotes apoptosis. Similarly, the SK1 inhibitor SKI-I decreases S1P, raises CER levels, induces G2-M arrest, and triggers apoptotic cell death, highlighting SK1 as a potential therapeutic target in melanoma [210].

TNF is a strong regulator of sphingolipid metabolism, triggering the SM-CER pathway through A-SMase activation [215]. In melanoma cells, SK1 and its product S1P are key components of TNF signaling [216]. High SK1 expression contributes to immune checkpoint inhibitors (like anti-PD-1 or anti-CTLA-4) resistance in melanoma, and its inhibition enhances treatment response, making SK1 a potential therapeutic target [217]. Emerging evidence suggests that sphingolipids play a role in melanoma dedifferentiation by regulating MITF. A-SMase promotes MAPK activation and MITF degradation, while AC, a direct MITF transcriptional target, is crucial for the phenotypic switch from invasive to proliferative melanoma cells [201,209]. A recent study assesses how TNF-driven changes in CER metabolism influence melanoma cell dedifferentiation. It demonstrates that TNF-induced inhibition of AC and increased glycosphingolipid production lead to CER metabolite accumulation and promote dedifferentiation [218]. Additionally, elevated glycosphingolipid levels were detected in the plasma of melanoma patients unresponsive to immune checkpoint inhibitors.

Ceramide can also be metabolized into more complex SLs, such as gangliosides. In human melanoma cells and tissues, levels of mono- and disialogangliosides, particularly GD3, are significantly elevated [202]. Overexpression of GD3 synthase in melanoma cells enhances proliferation and adhesion to the ECM, partly by recruiting integrins Via glycolipid-enriched microdomains [219,220]. GD3 promotes tumor growth by integrating pro-tumoral signals like HGF and c-MET [221]. GD3 correlates with increased c-Yes tyrosine kinase activity, which is higher in melanoma than in normal melanocytes [222]. Inhibiting c-Yes reduces the malignancy of GD3-positive melanoma cells, suggesting a potential therapeutic target [223]. Treatment with anti-GD3 antibody R24 decreases melanoma cell growth both in vitro and in vivo [224]. Similar effects are observed with GD2 synthase overexpression, which also enhances cell adhesion [225]. These findings highlight the distinct roles of gangliosides in melanoma progression: GD2 supports cell proliferation at both primary and metastatic sites, while GD3 promotes melanoma cell invasion, facilitating the transition to a metastatic phenotype.

Downregulation of AC leads to increased levels of S1P, which triggers the loss of E-cadherin in melanoma cells and raises ganglioside levels [209]. This promotes EMT and enhances adhesion to the extracellular matrix, contributing to the aggressive behavior of melanoma.

In addition, CER metabolism in melanoma is significantly affected by the downregulation of sphingomyelin synthase 1 (SMS1), the enzyme responsible for converting CER into SM [226]. This reduction does not lead to ceramide build-up, likely due to its diversion toward glucosylceramide (GlcCer) synthesis Via GlcCer synthase (GCS), resulting in increased GlcCer levels compared to SM in most melanoma cell lines [227]. Additionally, SM can be transformed into sphingosylphosphorylcholine (SPC), a bioactive lipid shown to drive melanoma progression by activating key signaling pathways, including ERK, MITF, and Akt/mTOR [228,229,230].

#### 3.3.3. Therapeutic Approaches Targeting Sphingolipids

Sphingolipids are promising therapeutic targets in melanoma [203]. Melanoma cells reduce ceramide to avoid cell death by altering enzymes like AC, SphK1, and GlCer synthase. Inhibiting these enzymes can restore ceramide levels and promote tumor cell death. Inhibition of AC with the stable inhibitor ARN14988 increases ceramide levels and sensitizes proliferative melanoma cells to various anticancer drugs [204]. Similarly, the chemotherapy agent dacarbazine has been shown to induce AC degradation, which contributes to its cytotoxic effect [231]. Exogenous ceramides can induce melanoma cell death, and their effectiveness is enhanced by structural modifications and delivery strategies [232]. They may also boost endogenous ceramide production Via the sphingosine-recycling pathway, further strengthening their anticancer action.

Treatment with exogenous ceramide, such as liposomal C6-ceramide, exhibited cytotoxic and antiproliferative effects in human melanoma cell lines by inducing caspase-dependent apoptosis Via activation of protein phosphatase 1 (PP1) [233]. Additionally, a brief 30-min treatment with nanoliposomal C6-ceramide reduced melanoma cell migration and tumor extravasation under shear stress, likely by lowering integrin affinity and activating the tumor-suppressive pathways PI3K and PKCζ [234].

Given the pivotal role of SK1/SK2 and S1P signaling in driving melanoma progression and resistance to therapy, various therapeutic approaches have been designed to inhibit these kinases or block S1P activity [232]. SPHK1 inhibitors (e.g., PF-543) and S1P receptor modulators (e.g., fingolimod) restore ceramide-mediated apoptosis and reduce T-cell exhaustion, improving the melanoma response to immunotherapies [235].

### 3.4. Oxylipins: Role in Melanoma and Therapeutic Targets

Oxylipins, produced from ω-3 and ω-6 PUFAs, are linked to inflammation, oxidative stress, and signaling [236]. They are generated either non-enzymatically or Via enzymes such as lipoxygenases (LOX), cyclooxygenases (COX), and cytochrome P450 (CYP450) [237]. Among them, prostaglandin E2 (PGE2) has been shown to promote cancer growth Via β-catenin signaling [238]. PGE2 supports cancer immune evasion by interfering with dendritic cells recruitment and suppressing type I interferon-driven tumor elimination [239,240].

High COX-2 expression is linked to poor prognosis in several cancers, including melanoma [241]. It promotes cell proliferation and invasion by activating key pathways involved in melanoma progression, such as MAPK, β-catenin, and EGFR/PI3K. However, COX-2 expression in human melanoma remains debated. In fact, some studies report no COX-2 expression in benign or malignant melanocytic tumors [242], others show that its expression levels increase alongside disease progression and metastasis [243,244,245,246]. A notable finding is that COX-2 expression exerts a substantial influence on melanoma’s metastatic potential, irrespective of the melanoma-related somatic mutations [247]. Additionally, prostaglandins synthesized by COX-2, such as PGE2 and PGF2α, promote melanogenesis and post-inflammatory pigmentation in melanoma cells. These PGs also enhance tumor growth by supporting cancer cell survival, migration, immune evasion, and angiogenesis [236].

Unlike its well-established role in promoting EMT and dedifferentiation in many other solid tumors, the COX-2/PGE2 signaling pathway in melanoma predominantly promotes a proliferative phenotype and thus a less dedifferentiated phenotype [248,249,250]. In melanoma cells high levels of MITF expression are associated with the proliferative state in presence of an active differentiation program, whereas low MITF expression is associated with an invasive, undifferentiated state characterized by reduced proliferative capacity [251]. Thus, evidence showing that inhibiting or silencing COX-2 reduces MITF expression and melanogenesis and decreases proliferation, migration, and invasion in vivo supports this distinction.

The COX-2/PGE2 pathway also contributes to creating a pro-inflammatory TME that favors tumor progression [250]. This process involves the recruitment of immune cells that contribute to tumor growth and aggressiveness. Further research is needed to clarify COX-2 exact role in melanoma.

Nonsteroidal anti-inflammatory drugs (NSAIDs) are being explored for melanoma chemoprevention due to their ability to inhibit COX enzymes. Aspirin and sulindac, both non-selective NSAIDs, have been studied in clinical trials for melanoma prevention. Notably, aspirin irreversibly inhibits COX by acetylation, and it preferentially targets COX-1 at low doses and COX-2 at higher doses [252]. Aspirin has demonstrated direct cytotoxicity against several human and murine melanoma cell lines [253]. Its action leads to reduced proliferation, decreased mitotic activity, and increased production of reactive oxygen species (ROS) in a dose- and time-dependent manner. Similarly, diclofenac, another NSAID, induced melanoma cell apoptosis Via ROS generation and mitochondrial dysfunction, supporting the anti-tumor potential of NSAIDs [254]. The specific COX-2 inhibitor celecoxib has been demonstrated to elicit apoptosis in melanoma cells through the induction of oxidative stress [255]. In addition, it has been demonstrated to reduce the expression of immune-suppressive molecules, such as PD-L1 [256]. These findings support celecoxib’s potential as a promising therapeutic adjuvant in the treatment of melanoma.

Other prostaglandins like thromboxane A2 (TXA2) and PGD2 have been investigated for their crucial role in cancer promotion. The decrease in TXA2, induced by inhibiting COX-1 with aspirin, reduced platelet–tumor interactions and recruitment of pro-metastatic immune cells [257]. In an experimental mouse metastasis model by the injection of syngeneic B16F10 melanoma tumor cells, it has been demonstrated that the anti-metastatic effect of COX-1 inhibition is limited to the early stages of metastasis [257]. In addition, the inhibition of COX-1 or TXA2 synthesis has been shown to impede the establishment of an intravascular metastatic and pre-metastatic niche. Conversely, PGD2 has shown tumor-suppressive effects by activating PPAR-γ, limiting tumor self-renewal, growth, and metastasis through inhibition of STAT3 signaling [258].

Although the pro-inflammatory and cancer-promoting effects of oxylipins generated by ω-6 PUFAs, such as arachidonic acid, are well documented, identifying the net effect of this heterogeneous group of substances, including those synthesized by ω-3 PUFAs (generally regarded as anti-inflammatory mediators), presents new challenges. Thus, oxylipins are synthesized simultaneously from different PUFAs and exert diverse functions at the cellular level. However, the mechanisms by which cells integrate and respond to these multiple signals are not well understood. Therefore, rather than examining oxylipin synthesis as isolated compounds, it should be examined as comprehensive profiles that reflect the physiological state of the organisms under study.

Recent advances in mass spectrometry-based approaches for oxylipin profiling have enabled more detailed research on oxylipin biology in pathological processes. These advances have also highlighted the potential of oxylipins as biomarkers for a wide range of diseases [259]. An additional challenge is the pronounced heterogeneity of tumors, including melanoma. Numerous studies indicate substantial metabolic variability, especially in lipid profiles, both among melanoma subtypes and across individual patients. Since oxylipins are key mediators of inflammation and the immune response, analyzing their profile in a blood sample, specifically the difference between pro-inflammatory and anti-inflammatory oxylipins, may reveal metabolic signatures that correlate with the efficacy of immunotherapy in melanoma [260].

Nowadays, HPLC-MS lipidomic platforms are used for routine workflows that enable the quantitative profiling of up to 150–200 oxylipins from all pathways and substrate PUFAs [259]. However, because this approach cannot resolve isomers, chiral separation methods are sometimes required to distinguish enzymatic from free-radical–derived oxylipins. Although ELISA kits have long been employed to detect oxylipins such as PGs, their limited specificity and tendency to overestimate concentrations restrict their reliability, and they are often used only as indicators of pathway activity rather than for precise quantification. Analytical choices can have a profound influence on the resulting oxylipin profiles, making validation studies, standardization, and regular literature updates essential for accurate biological interpretation [259]. Many circulating oxylipins lack cell or tissue specificity, arise from multiple metabolic pathways, and include poorly characterized metabolites. Harmonized analytical procedures are therefore needed to ensure high-quality, comparable data.

## 4. Lipidomics in Melanoma Research

### 4.1. Technologies

Developing innovative analytical platforms is essential for identifying specific pathological lipid signatures of melanoma using lipidomic approaches.

Mass spectrometry (MS) is the key technology in lipidomics due to its exceptional sensitivity and ability to resolve complex lipid structures. Its versatility stems from the wide range of ion sources and analyzers, enabling tailored strategies for lipid analysis [261]. One such method is shotgun lipidomics, which involves direct infusion of lipid extracts into high-resolution MS for accurate mass detection and differentiation of similar compounds. Instruments like triple quadrupole (QqQ), Orbitrap, and quadrupole-time of flight (QTOF) enhance lipid identification and support large-scale, high-throughput lipid profiling across diverse lipid classes [262].

To overcome limitations of shotgun lipidomics, such as ion suppression, artifacts, and difficulty distinguishing isomeric/isobaric species, a separation step prior to MS is often introduced [263]. Liquid chromatography (LC) is the most widely used technique for this, offering better resolution than gas chromatography. LC-MS enables lipid separation by polarity or headgroup, improving quantification and structural identification. Reversed-phase LC (RPLC) is most common, while normal-phase LC (NPLC) and hydrophilic interaction chromatography (HILIC) are used for more polar compounds. Recent advances like ultra-high-performance LC (UHPLC) and ion mobility spectrometry (IMS-MS) enhance separation, with IMS distinguishing isomeric species by their mobility in a gas under an electric field [261,264]. Supercritical fluid chromatography (SFC), using CO_2_ as the mobile phase, offers rapid, efficient separation across a range of lipid polarities, though its adoption is limited due to technical constraints like pressure limits [265]. Ultimately, the choice of method depends on the lipid class of interest and sample characteristics [266].

MS, due to its high specificity, sensitivity, and throughput, is considered a gold-standard tool for analyzing lipid composition and distribution, even at the single-cell level. This enables detailed investigation of cancer cell heterogeneity and phenotypic plasticity. The advancement of single-cell lipidomics allows identification of unique lipid profiles in individual tumor cells, offering deeper insights into tumor complexity and uncovering lipid pathways relevant to diagnosis, progression, and prognosis [267,268,269]. However, a key challenge is isolating cells in their native state without inducing stress or death, which could alter their lipid profiles. To address this, capillary and microfluidic technologies are being developed to improve single-cell handling and preserve native lipid content during analysis [270,271,272,273]. Single-cell lipidomics faces key challenges, including low lipid abundance, potential contamination, and ion suppression during MS analysis [264,274]. Despite these limitations, techniques like mass spectrometry imaging (MSI) offer powerful solutions for studying spatial lipid distribution in tissues while preserving native biological context. MSI combines ionization methods, such as desorption electrospray ionization (DESI), secondary ion MS (SIMS), or matrix-assisted laser desorption/ionization (MALDI), with high-resolution MS to map lipid molecules across tissue sections with near single-cell precision [261]. Unlike traditional methods, MSI avoids extraction, purification, or labeling, reducing artifacts and maintaining lipid integrity. This approach enables the visualization of lipid heterogeneity and their spatial relationship within the tumor microenvironment, offering critical insights into tumor biology, especially intratumoral heterogeneity, and its links to pathological conditions like cancer. Furthermore, MSI is evolving into a powerful tool for “molecular histology,” offering high-resolution spatial maps of lipids that could transform clinical diagnostics [275]. Innovations like MALDI-2 and AFADESI enhance spatial resolution and ionization efficiency, even enabling subcellular lipid analysis [276,277,278]. However, the complexity and volume of data generated by lipidomics require robust bioinformatics tools. Databases and specialized software platforms are essential for lipid identification, spectral processing, pathway mapping, and statistical interpretation. While these tools vary in function and performance, they collectively support accurate and meaningful analysis of lipidomic data. For detailed insights into such platforms, the reader is directed to more focused literature [279,280,281,282,283].

Despite the availability of dedicated lipidomic software tools, reliable lipid annotation and structural characterization remain challenging due to the extensive structural diversity of lipids and the complexity of mass spectrometry data. In this context, Artificial Intelligence (AI), particularly Machine Learning (ML), has gained increasing attention as a complementary strategy to support lipidomic data analysis. ML relies on algorithms capable of learning patterns from large and complex datasets to build predictive and classification models that can be applied to new, unseen data [284]. Several ML algorithms, including random forest, which combine multiple decision trees for robust predictions, have been applied to support lipid annotation, chromatographic retention time prediction, and MS/MS spectrum interpretation, with the aim of improving annotation consistency and lipid coverage compared to conventional rule-based workflows [285,286,287]. In parallel, deep learning-based approaches, which rely on multilayer neural network architectures capable of automatically extracting complex and non-linear features from high-dimensional data, are being explored to improve lipidomics annotation rates [288]. Specific tools illustrate these advances. LipidOA is a ML-based tool which focuses on structural-level annotation, integrating ML with prior biochemical knowledge, such as Paternò–Büchi (PB) reactions, to model spectral feature relationships for successfully locating the exact position of carbon–carbon double bonds in a specific fatty acyl chain [286]. In contrast, MS2Lipid is designed for class-level lipid annotation, using spectral features to classify lipid subclasses even when full structural information is unavailable, thus accelerating annotation of previously unknown lipids [287]. These approaches enhance annotation confidence, reproducibility, and facilitate comparative analyses of complex lipidomic datasets. Moreover, their application reduces manual curation and improves consistency across large-scale lipidomic datasets. Beyond identification, ML-based methods aim to detect specific features and relationships among different lipid species observed, making them essential for uncovering patterns and associations that are challenging to identify using traditional statistical methods. These capabilities are crucial for investigating disease mechanisms, discovering biomarkers, and stratifying patients according to disease progression. Consequently, ML has a wide range of applications in cancer research and diagnostics, offering significant potential for enhancing our understanding of melanoma and improving therapeutic strategies [289,290,291].

### 4.2. Transfer of Laboratory Data into Clinical Practice

The translation of academic lipidomics research into clinically applicable diagnostic tools is a complex and lengthy process. This challenge stems not only from technical limitations, such as lipid fragmentation, low abundance, and lack of standardization, but also from significant administrative and regulatory hurdles. These include stringent validation requirements, compliance with healthcare regulations, and the need for robust, reproducible protocols before integration into clinical workflows. However, lipidomics clearly shows the potential to differentiate between healthy and tumoral tissue for melanoma.

Mass spectrometry-based lipidomics (DI-MS, UHPLC-MS) has identified distinct lipid alterations in melanoma cells, including a selective accumulation of PCs, particularly ether-linked PCs, unlike benign nevi, which show reduced PC levels [189]. While sphingomyelins (SMs) are generally decreased in melanoma cells, this does not correlate with metastatic potential [189,292]. Instead, melanoma progression is marked by elevated phosphatidylinositol (PI) species, especially saturated forms like PI 16:0/18:0 and PI 18:0/18:0, suggesting their value as biomarkers of aggressiveness [189,292].

Lipidomic profiling has been extended to melanoma-derived exosomes, which may contribute to tumor motility and invasiveness [293,294]. MALDI-TOF/MS analysis shows that these exosomes are enriched in SMs and have reduced PI content relative to parental cells. However, lipid profiles of exosomes did not differ significantly between low and highly metastatic melanoma cells [295]. Collectively, PCs, PIs, and SMs emerge as promising biomarkers for melanoma detection and monitoring.

Distinguishing primary invasive melanoma from metastasis remains a diagnostic challenge. Spatial lipidomics using TOF-SIMS revealed clear lipidomic differences between primary and metastatic melanoma, including elevated phosphatidylethanolamines and GM3 gangliosides in metastases [296]. Variations in phospholipid and GM3 chain profiles may further differentiate in-transit from distant metastases. These localized lipid alterations have the potential to serve as biomarkers for melanoma progression, supporting diagnosis and therapeutic development.

Imaging mass spectrometry techniques have also proven valuable for histological differentiation between healthy skin, nevi and melanoma [188,297]. This capability to distinguish tissue types based on lipid composition has led to the filing of a patent, highlighting its potential for clinical diagnostic applications [42].

Growing evidence supports the role of serum and plasma biomarkers in improving melanoma diagnosis. Plasma extracellular vesicles (EVs), acting as “liquid biopsies,” reflect tumor status and are emerging as promising non-invasive biomarkers. A recent study integrating proteomic and metabolomic profiling of plasma-derived EVs identified key lipid metabolites, especially PCs and a lysophosphatidylcholine (lysoPC), that differentiate melanoma patients from healthy controls and distinguish primary from metastatic disease [298]. These changes, such as increased PC aa C38:0 and reduced PC ae C34:3 and lysoPC a C18:2, suggest heightened phospholipase A2 activity associated with tumor invasiveness.

Additionally, untargeted serum metabolomics across two melanoma patient cohorts highlighted consistent alterations in lipid, organic acid, and amino acid metabolism. Enhanced lipid metabolism emerged as a hallmark of melanoma, supporting rapid proliferation and metastasis [299]. Together, these findings emphasize the diagnostic and prognostic value of lipid-based biomarkers in melanoma and their potential to inform clinical decision-making.

Melanoma prognosis significantly improves with early detection, driving interest in blood-based biomarkers. Using untargeted metabolomics Via LC-HRMS, a study identified ten serum metabolites, mainly lipids, altered in stage I melanoma patients, most of which were reduced compared to healthy controls [300]. A predictive model based on three key metabolites showed high diagnostic accuracy and was validated in an independent cohort, supporting the potential of serum lipidomics for early melanoma detection.

Further research explored the relationship between plasma lipid profiles and key clinical features of primary melanoma, including Breslow thickness and ulceration [301]. Using the Lipidyzer™ platform, which profiles over 1100 lipid species across 13 classes, specific lipid changes were found to correlate with tumor thickness and ulceration. A four-lipid model (CE (20:5), LCER (24:1), PE (P18:1/18:1), LPE (18:2)) predicted Breslow depth, while two lipids (FFA (16:0), PC (15:0/18:1)) were linked to ulceration.

In a larger cohort of 151 melanoma patients (83 non-metastatic, 68 metastatic), Lipidyzer™ analysis revealed a global reduction in plasma lipids in metastatic patients, especially in FFAs and LCERs [302]. Three lipids (CE (12:0), FFA (24:1), TG47:2-FA (16:1) outperformed LDH and S100B in predicting metastasis. Elevated levels of five LPCs were associated with lymph node metastasis, and seventeen lipids were linked to patient survival. These studies underscore the diagnostic and prognostic potential of lipidomic biomarkers in melanoma progression and metastasis.

Lipidomic analyses have revealed key insights into melanoma progression, therapy resistance, and immune response. Dysregulation in lipid metabolism, particularly involving FASN and DHCR24, correlates with melanoma status and plasma lipid levels. Advanced melanoma patients displayed altered FA derivatives and SLs, with targeted profiling identifying elevated levels of dihydro-CERs, CERs, SMs, and GM3 ganglioside. Stratification by response to BRAF/MEK inhibitors showed that levels of specific fatty acids (FA16:0, FA18:0, FA18:1) were linked to better prognosis, suggesting lipid profiles could serve as indicators of therapeutic response [303].

**Figure 2:**
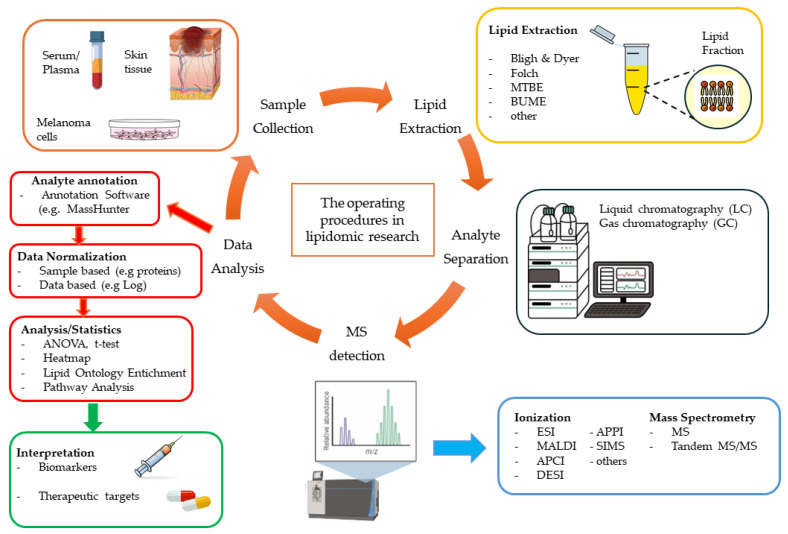
Schematic representation of the operating procedures in lipidomic research from the sample collection to the data analysis. Extraction Solvents: BUME (Butanol-Methanol); MTBE (Methyl Tert-Butyl Ether); Ionization Sources: APCI (Atmospheric Pressure Chemical Ionization); APPI (Atmospheric Pressure Photoionization); DESI (Desorption Electrospray Ionization); ESI (Electrospray Ionization); MALDI (matrix-assisted laser desorption/ionization); SIMS (Secondary Ion Mass Spectrometry).

Additionally, targeted metabolomics of pre-treatment serum from patients receiving immune checkpoint inhibitors (ICI) revealed subtype-specific metabolic signatures [304]. Uveal and mucosal melanomas differed significantly from cutaneous melanoma, especially in tryptophan-kynurenine metabolism, sphingomyelin composition (e.g., SM-d18:1/22:1), and elevated spermine levels—markers associated with ICI resistance. Cutaneous melanoma patients with lower spermine had better survival outcomes. These findings support the potential of metabolomic and lipidomic profiling to identify predictive biomarkers for treatment stratification and to understand mechanisms underlying ICI resistance. A schematic representation of the operating procedures in lipidomic research from the sample collection to the data analysis is reported in Figure 2.

## 5. Conclusions

Advances in lipidomics technologies, particularly mass spectrometry-based approaches, have enabled the comprehensive characterization of lipid alterations in melanoma at bulk, spatial, and single-cell levels. Lipidomics provides an unprecedented opportunity to identify diagnostic and prognostic lipid signatures, uncover mechanisms of therapy resistance, and stratify patients based on metabolic vulnerabilities. Moreover, lipid-derived biomarkers in tissues and biofluids hold promise for improving early detection, disease monitoring, and treatment response assessment through minimally invasive approaches.

However, several challenges still hinder the clinical application of lipidomics in melanoma. Despite advancements in MS-based lipidomics, achieving a truly comprehensive characterization of the lipidome remains challenging. The majority of current methods can detect only a fraction of the total lipid species, limiting global lipidome coverage. Certain lipid classes, such as eicosanoids and oxysterols, often require separate targeted analyses with analyte enrichment and larger sample volumes, unlike more abundant lipids like glycerophospholipids or sphingolipids. Moreover, comprehensive lipidome analysis is limited by the presence of isomeric/isobaric lipid species, which require chromatographic separation, something not achievable with shotgun approaches. These limitations reduce the information obtainable from a single lipidomics method. However, advances in MS sensitivity, chromatographic techniques, and ionization methods (e.g., nano-LC–MS, ion mobility) may eventually enable detection of even low-abundance lipids from very small samples like biopsies.

One longstanding issue is the need for standardized normalization and quality control procedures to ensure reliable data interpretation [305]. Encouragingly, ongoing efforts within the lipidomics community have brought the field closer to achieving consensus on these critical methodological standards [306,307,308,309]. Most lipidomic studies in cancer, including melanoma, currently use machine learning, but deep learning is emerging as the next step [310]. While deep learning has been applied in cancer research, it is not yet common in lipidomics [311].

Current methods are effective for distinguishing cancer from healthy tissue, especially in imaging lipidomics. Melanoma is well-suited for such approaches due to easy biopsy access, and lipidomics imaging may help overcome the lack of reliable antigenic markers in diagnosis [188,312]. Bulk lipidomics provides an overall lipid profile of a tissue or sample extract but overlooks spatial variations caused by different cell types within the tissue. To address this limitation, spatial lipidomics is employed, allowing for the visualization and analysis of lipid distribution within the tissue, thereby revealing regional and cellular heterogeneity. This approach might help the clinician in making prognoses and therapy selection.

Recent advancements in lipid analysis techniques have enabled comprehensive investigations of the lipidome in biological samples, driving research into lipid changes in patient biofluids, such as serum or plasma, to better understand cancer-related metabolic alterations.

In order to use liquid biopsies to diagnose melanoma, lipidomic strategies must be optimized to identify biomarkers that can distinguish between healthy individuals and melanoma patients, going beyond the current capabilities. However, although liquid biopsy-based lipidomics only distinguishes between healthy and diseased individuals and does not allow for disease stratification, it can be considered a valid follow-up technique as it makes post-intervention control tests easier, faster and less invasive.

Combining multi-omics approaches can overcome the limitations of single-approach strategies. Integrating lipidomics with genomic, transcriptomic, and metabolomic data will be essential to fully elucidate melanoma metabolic heterogeneity and to translate lipid-targeted interventions into clinical practice. This multi-omics approach enables deeper insights into complex processes that single-omics studies may miss. Integration strategies include co-expression, pathway, and network analyses, each suited to specific research goals [313]. As technologies and computational tools evolve, omics integration is becoming increasingly essential for uncovering molecular mechanisms and advancing precision medicine.

In certain cancers, such as prostate cancer, integrative models that combine data from multiple omic layers have shown improved accuracy in characterizing tumor subtypes and predicting patient outcomes [314]. While such multi-omic approaches are still in their early stages and have yet to be widely applied to melanoma, they hold significant potential. As the field progresses, it is likely that these integrative strategies will rely heavily on machine learning to interpret complex datasets, ultimately providing a more comprehensive understanding of melanoma biology and informing more precise treatment strategies.

In conclusion, lipidomics shows great potential in translational melanoma research. The identification of melanoma-specific lipid biomarkers can support the early diagnosis and prognosis, as well as the development of personalized treatment strategies. In the future, lipidomics may become part of routine clinical practice, enabling non-invasive monitoring of disease progression and treatment response through liquid biopsies.

## Data Availability

No new data were created or analyzed in this study. Data sharing is not applicable to this article.

## Abbreviations

AC (acid ceramidase); AI (Artificial Intelligence); ACATs (acyl-CoA-cholesterol acyltransferases); ACC (acetyl-CoA carboxylase); ACLY (ATP citrate lyase); ACSL4 (acyl-CoA synthetase long-chain family member 4); AFADESI (airflow-assisted desorption electrospray ionization); AKT (Protein Kinase B); AMPK (AMP-activated kinase); APCI (Atmospheric Pressure Chemical Ionization); APPI (Atmospheric Pressure Photoionization); ARF (Alternate reading frame); ATX (autotaxin); BAP1 (BRCA1 associated protein 1); BRAF (B-Raf proto-oncogene, serine/threonine kinase); BUME (Butanol-Methanol); CACT (carnitine acylcarnitine translocase); CDK4 (Cyclin-dependent kinase 4); CDKN2A (Cyclin-dependent kinase inhibitor 2A); CEs (cholesterol esters); CER (ceramide); CERS (ceramide synthase); CH (cholesterol); CM (cutaneous melanoma); COX (cyclooxygenase); CPT1 (carnitine palmitoyltransferase 1); CSCs (cancer stem cells); CTLA-4 (Cytotoxic T-lymphocyte associated protein 4); CYP450 (cytochrome P450); DAG (diacylglycerol); DESI (desorption electrospray ionization); DI-MS (direct infusion-MS); DNL (de novo lipogenesis); ECM (extracellular matrix); EGF (Epidermal Growth Factor); ELOVLs (fatty acid elongases); EMT (epithelia-mesenchymal transition); ER (endoplasmic reticulum); ERK (Extracellular signal-Regulated Kinase); ESI (Electrospray Ionization); EV (extracellular vesicles); FAs (fatty acids); FABP (FA binding proteins); FAO (Fatty acid oxidation); FASN (FA synthase); FAT/CD36 (FA translocase); FATP CD36 (FA transport proteins); FFAs (free fatty acids); GCS (GlcCer synthase); GD (disialogangliosides); GlcCer (glucosylceramide);GLUT1 (Glucose Transporter type 1); GPLs (Glycerophospholipids); HER2 (Human Epidermal growth factor Receptor 2); HGF (Hepatocyte Growth Factor); HIF-1 (Hypoxia-Inducible Factor 1); HILIC (hydrophilic interaction chromatography); HMGCR (3-hydroxy-3-methyl-glutaryl-coenzyme A reductase); IMS–MS (ion mobility spectrometry); KGF (Keratinocyte Growth Factor); KIT (KIT proto-oncogene receptor tyrosine kinase); LC (liquid chromatography); LDs (lipid droplets); LDH (Lactate Dehydrogenase); LDLR (low-density lipoprotein receptor); LOX (lipoxygenase); LPA (lysophosphatidic acid); LPC (lysophosphatidylcholine); LPCAT (Lysophosphatidylcholine acyltransferase); LXRs (liver X receptors); LysoPC (lysophosphatidylcholine); MAG (monoacylglycerol); MALDI (matrix-assisted laser desorption/ionization); MALDI-2 (two-step laser-based method); MAPK (Mitogen-Activated Protein Kinase); MC1R (melanocortin-1 receptor); MCD (methyl-cyclodextrin); MCT1/4 (Monocarboxylate Transporters 1 and 4); MEK (Mitogen-activated Extracellular signal-regulated Kinase); c-MET (Mesenchymal–Epithelial Transition factor); MITF (Microphthalmia-associated transcription factor); ML (Machine Learning);MS (Mass spectrometry); MSI (mass spectrometry imaging); MTBE (Methyl Tert-Butyl Ether); MUFAs (monounsaturated fatty acids); c-MYC (MYC Proto-Oncogene); NADPH (nicotinamide adenine dinucleotide phosphate); NANOG (Homeobox protein NANOG); NF1 (Neurofibromin 1); NPLC (normal-phase LC); NRAS (Neuroblastoma RAS viral oncogene homolog); NSAIDs (Nonsteroidal anti-inflammatory drugs); OCT4 (Octamer-binding transcription factor 4); PC (phosphatidylcholine); PD-1 (Programmed cell death protein 1); PE (phosphatidylethanolamine); PG (phosphatidylglycerol); PA (phosphatidic acid); PI (phosphatidylinositol); PI3K (Phosphatidylinositol 3-kinase); PGC1α (Peroxisome proliferator-activated receptor gamma coactivator 1-alpha); PGPX4 (glutathione peroxidase 4); PLs (phospholipids); PLA2 (phospholipase A2); PP1 (protein phosphatase 1); PPAR-α (peroxisome proliferator-activated receptor alpha); PGE2 (prostaglandin E2); PPAR-γ (peroxisome proliferator-activated receptor gamma); PS (phosphatidylserine); PTEN (Phosphatase and tensin homolog); PGs (prostaglandins); PUFAs (polyunsaturated fatty acids); QqQ (triple quadrupole); QTOF (quadrupole-time of flight); ROS (reactive oxygen species); RPLC (Reversed-phase LC); S1P (sphingosine-1-phosphate); SCD (stearoyl-CoA desaturase); SFC (Supercritical fluid chromatography); SIMS (secondary ion MS); SLs (sphingolipids); SM (sphingomyelin); SMS1 (sphingomyelin synthase 1); ACAT (acyl-CoA:cholesterol acyltransferase); SPL (S1P lyase); SRY (Sex-determining Region Y)-box 2); SPC (sphingosylphosphorylcholine); SPH (sphingosine); SphK1 (Sphingosine kinase 1); SREBP1 (sterol regulatory element-binding protein-1); STAT3 (Signal Transducer and Activator of Transcription 3); TCA (tricarboxylic acid); TGs (triacylglycerides); TME (tumor microenvironment); mTOR (Mechanistic Target of Rapamycin); TAGs (triacylglycerols); TP53 (Tumor protein p53); TXA2 (thromboxane A2); UHPLC (ultra-high-performance LC); UV (ultraviolet light).
